# 7 Tesla MRI of the *ex vivo* human brain at 100 micron resolution

**DOI:** 10.1038/s41597-019-0254-8

**Published:** 2019-10-30

**Authors:** Brian L. Edlow, Azma Mareyam, Andreas Horn, Jonathan R. Polimeni, Thomas Witzel, M. Dylan Tisdall, Jean C. Augustinack, Jason P. Stockmann, Bram R. Diamond, Allison Stevens, Lee S. Tirrell, Rebecca D. Folkerth, Lawrence L. Wald, Bruce Fischl, Andre van der Kouwe

**Affiliations:** 1 Center for Neurotechnology and Neurorecovery, Massachusetts General Hospital, Department of Neurology, Boston, MA 02114 USA; 2 Athinoula A. Martinos Center for Biomedical Imaging, Massachusetts General Hospital, Department of Radiology, Charlestown, MA 02129 USA; 3 Movement Disorders & Neuromodulation Section, Department for Neurology, Charité – University Medicine Berlin, Berlin, Germany; 4 Radiology, Perelman School of Medicine, University of Pennsylvania, Philadelphia, PA 19104 USA; 5 City of New York Office of the Chief Medical Examiner, and New York University School of Medicine, New York, NY 10016 USA

**Keywords:** Brain, Brain injuries, Magnetic resonance imaging, Brain imaging

## Abstract

We present an ultra-high resolution MRI dataset of an *ex vivo* human brain specimen. The brain specimen was donated by a 58-year-old woman who had no history of neurological disease and died of non-neurological causes. After fixation in 10% formalin, the specimen was imaged on a 7 Tesla MRI scanner at 100 µm isotropic resolution using a custom-built 31-channel receive array coil. Single-echo multi-flip Fast Low-Angle SHot (FLASH) data were acquired over 100 hours of scan time (25 hours per flip angle), allowing derivation of synthesized FLASH volumes. This dataset provides an unprecedented view of the three-dimensional neuroanatomy of the human brain. To optimize the utility of this resource, we warped the dataset into standard stereotactic space. We now distribute the dataset in both native space and stereotactic space to the academic community via multiple platforms. We envision that this dataset will have a broad range of investigational, educational, and clinical applications that will advance understanding of human brain anatomy in health and disease.

## Abstract (summary)

**Table:** 

Measurement(s)	nuclear magnetic resonance assay
Technology Type(s)	MRI Scanner
Factor Type(s)	flip angle
Sample Characteristic - Organism	Homo sapiens

Machine-accessible metadata file describing the reported data: 10.6084/m9.figshare.9958688

## Background & Summary

Postmortem *ex vivo* MRI provides significant advantages over *in vivo* MRI for visualizing the microstructural neuroanatomy of the human brain. Whereas *in vivo* MRI acquisitions are constrained by time (i.e. ~hours) and affected by motion, *ex vivo* MRI can be performed without time constraints (i.e. ~days) and without cardiorespiratory or head motion. The resultant advantages for characterizing neuroanatomy at microscale are particularly important for identifying cortical layers and subcortical nuclei^1–5^, which are difficult to visualize even in the highest-resolution *in vivo* MRI datasets^6,7^. *Ex vivo* MRI also provides advantages over histological methods that are associated with distortion and tearing of human brain tissue during fixation, embedding, and slide-mounting.

As the field of *ex vivo* MRI has developed over the past two decades, several laboratories have focused on imaging blocks of tissue from human brain specimens using small-bore scanners^2,8^ and specialized receive coils^9–11^. This approach allows for spatial resolutions of up to 35–75 microns for analyses of specific neuroanatomic regions^9,11–13^. However, ultra-high resolution imaging of whole human brain specimens at high magnetic field strengths has been far more challenging, due to the need for multi-channel receive coils and large-bore clinical scanners that can accommodate a whole-brain specimen. Whole-brain imaging is required to observe neuroanatomic relationships across distant brain regions, as well as to provide a complete view of human neuroanatomy in standard stereotactic space.

Here, we report the results of a multidisciplinary effort to image a whole human brain specimen *ex vivo* at an unprecedented spatial resolution of 100 µm isotropic. Central to this effort was the construction of an integrated system consisting of a custom-built 31-channel receive array coil and volume transmit coil, which was designed to accommodate and tightly enclose an *ex vivo* human brain^14^. The scans were performed on a 7 Tesla whole-body human MRI scanner using four single-echo spoiled gradient-recalled echo (SPGR/GRE) or Fast Low-Angle SHot (FLASH) sequences. We used varying flip-angles (FA15°, FA20°, FA25°, FA30°) to generate multiple synthesized volumes, each of which is a recomputed image that provides a different tissue contrast. The scans, performed over ~100 hours (~25 hours per FA), generated an ~8 TB dataset (~2 TB per flip angle) that required custom-built computational tools for offline MRI reconstruction and creation of the synthesized volumes. Offline MRI reconstruction considerably reduces the data amount. We release the resulting FA25° acquisition, as well as the synthesized FLASH25 volume here, both in native space and coregistered to standard stereotactic space, for use by the academic community. We envision a broad range of investigational, educational, and clinical applications for this dataset that have the potential to advance understanding of human brain anatomy in health and disease.

## Methods

### Specimen acquisition and processing

A 58-year-old woman with a history of lymphoma and stem cell transplantation, but no history of neurological or psychiatric disease, died in a medical intensive care unit. She was initially admitted to the hospital for fevers, chills, and fatigue, and then was transferred to the intensive care unit for hypoxic respiratory failure requiring mechanical ventilation. Her hospital course was also notable for a deep venous thrombosis and a pulmonary embolism. The cause of her death on hospital day 15 was determined to be hypoxic respiratory failure due to viral pneumonia. At the time of her death, her surrogate decision-maker provided written informed consent for a clinical autopsy and for donation of her brain for research, as part of a protocol approved by our Institutional Review Board.

**Fig. 1:**
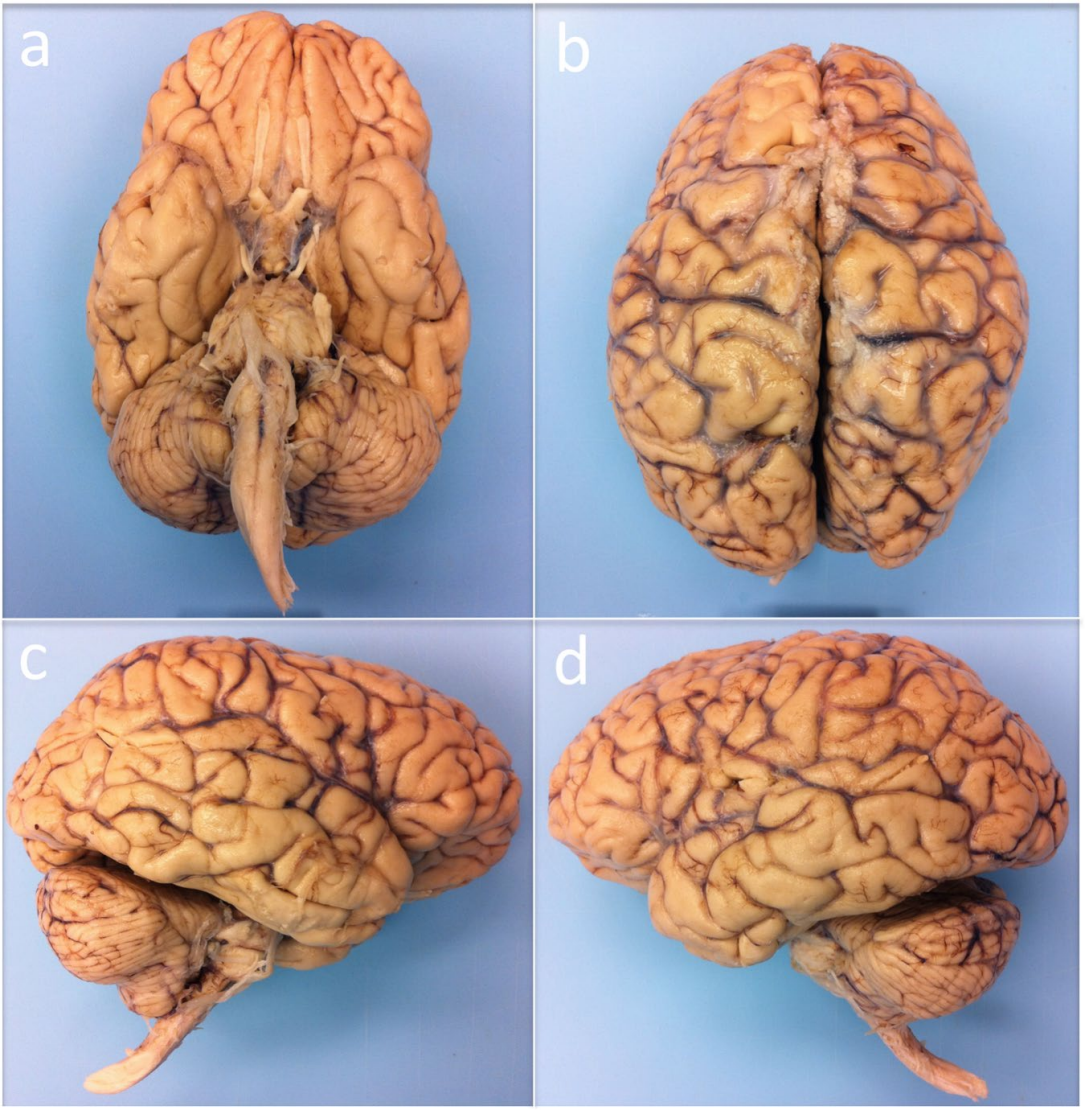
Human brain specimen. The human brain specimen that underwent *ex vivo* MRI is shown from inferior (**a**), superior (**b**), right lateral (**c**) and left lateral (**d**) perspectives. Gross pathological examination of the brain was normal.

At autopsy, her fresh brain weighed 1,210 grams (normal range = 1,200 to 1,500 grams). The brain was fixed in 10% formalin 14 hours after death. Gross examination revealed a normal brain (Fig. 1), without evidence of mass lesions or cerebrovascular disease. To ensure adequate fixation and prevent specimen flattening (which can prevent specimens from fitting into custom *ex vivo* MRI coils), we followed a series of standard specimen processing procedures, as previously described^15^.

### Specimen preparation for scanning

After remaining in fixative for 35 months, the brain specimen was transferred to Fomblin Y LVAC 06/6 (perfluoropolyether, Solvay Specialty Polymers USA, LLC, West Deptford, NJ), which is invisible to MRI and reduces magnetic susceptibility artifacts. The specimen, immersed in Fomblin, was then secured inside a custom-built, air-tight brain holder made of rugged urethane^16^. The brain holder contains degassing ports for removal of air bubbles, which further reduces magnetic susceptibility artifacts.

### Construction of a receive array coil and transmit volume coil for *ex vivo* imaging of the whole human brain

**Fig. 2:**
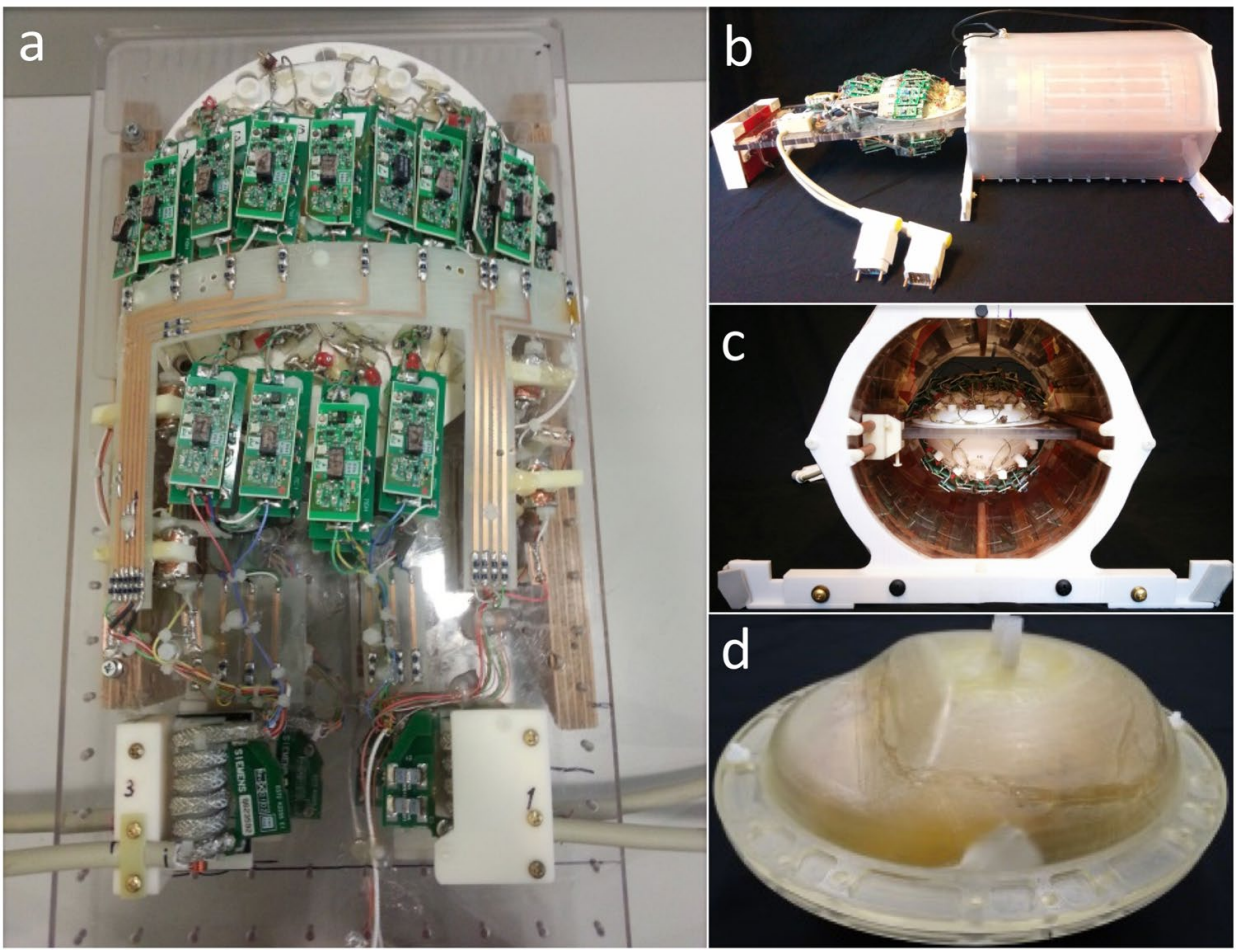
Receive array coil and transmit volume coil for *ex vivo* imaging of the whole human brain. (**a**) The 31-channel receive array has 15 elements on the top half (with a diameter of 5.5 cm) and 16 on the bottom half (with a diameter of 8.5 cm), each made of 16 AWG wire loops with four or five evenly spaced capacitors. All elements are tuned to 297.2 MHz. (**c**) The coil former has slightly larger dimensions than the brain holder, which slides inside a volume coil (**b**). (**d**) A custom air-tight brain holder was designed to conform to the shape of a whole human brain. The brain holder is an oblate spheroid container (16 × 19 cm) with degassing ports that are used to apply a vacuum suction, thereby reducing air bubbles in the specimen and surrounding fomblin solution.

We built a receive coil apparatus consisting of a 31-channel surface coil loop array with two halves. The apparatus was fabricated using a 3D printer of slightly larger dimensions than the brain holder, which slides inside the single-channel birdcage volume transmit coil (Fig. 2). The brain holder is an oblate spheroid (16 × 19 cm) that conforms to the shape of a whole brain (cerebral hemispheres + cerebellum + brainstem)^16^ (Fig. 2d). It is made of two separate halves that can be sealed together with a silicone gasket after packing the brain inside. This holder must also withstand the degassing process when under vacuum pressure. Degassing is performed in three steps: 1) introducing vacuum suction into the container with the brain inside, which allows the bubbles to expand under decreased pressure and exit tissue cavities; 2) opening the valve to fill the holder with fomblin and then sealing off the fill valve; and 3) continuation of vacuum suction with low-amplitude vibration of the holder for 2–6 hours. The vibration facilitates the removal of bubbles from tissue cavities. All three steps are performed inside a fume hood.

The coil former (Fig. 2c) consists of two halves and encloses the brain holder. The receive array coil consists of 31 detectors (Fig. 2a), with 15 elements on the top half (diameter = 5.5 cm) and 16 on the bottom half (diameter = 8.5 cm). Coil elements were constructed using 16 AWG wire loops^17^, each with four or five evenly spaced capacitors (Supplementary Fig. 1). All elements were tuned to 297.2 MHz and matched to a loaded impedance of 75 Ω to minimize preamplifier noise. Preamplifier decoupling was achieved with a cable length of 6 cm. Preamplifiers were placed directly on the coil elements, yielding a substantial reduction in cable losses compared to a previous 30-channel *ex vivo* brain array^18^. The active detuning circuit was formed across the match capacitor using an inductor and PIN diode.

Tuning, matching, and decoupling of neighboring elements was optimized on the bench with a brain sample immersed in periodate-lysine-paraformaldehyde (PLP) solution. Because coil loading varies with the fixative used, the coil must be tuned and matched on the bench using a brain sample with the correct fixative. (For example, testing can be performed with a brain sample immersed in PLP or formalin, but not the regular loading phantom comprised of water and salt). Loops tuned/matched on PLP showed unloaded-to-loaded quality factor ratio (Q-ratio) of Q_UL_/Q_L_ = 210/20 = 10.5, corresponding to an equivalent noise resistance of 11 ohms for the loaded coil (Q = wL/R). By contrast, formalin is a less lossy fixative, giving a coil Q-ratio of Q_UL_/Q_L_ = 210/60 = 3.5, corresponding to an equivalent noise resistance of 4 ohms.

A shielded detunable volume coil (Fig. 2) was built for excitation, with the following parameters and features: band-pass birdcage, diameter 26.7 cm, and an extended length of 32 cm to accommodate brain samples of larger dimensions. For the detuning circuit we used diodes in every leg of the birdcage. These diodes are powered with the high-power chokes, which can withstand high voltage and short duration inversion pulses.

In summary, this coil system incorporates an improved mechanical design, preamps mounted at the coil detectors, and an extended transmit coil design capable of producing high-power pulses.

### 7 Tesla MRI data acquisition

**Fig. 3:**
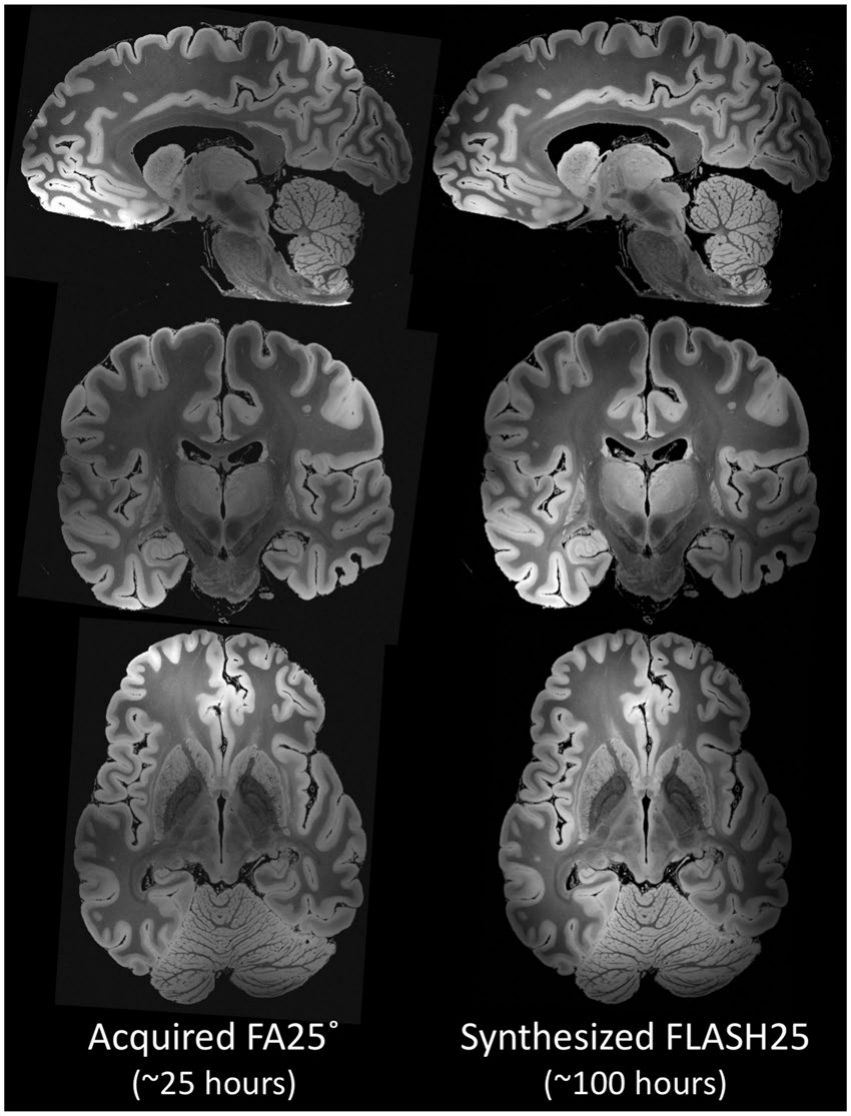
Comparison of FA25° acquisition and synthesized FLASH25 volume. Representative images from the FA25° acquisition (left column) and the synthesized FLASH25 volume (right column) are displayed in the sagittal (top row), coronal (middle row) and axial (bottom row) planes. These images provide a qualitative comparison of the respective signal-to-noise properties of the FA25° acquisition (~25 hours) and the synthesized FLASH25 volume (~100 hours). All images are shown in radiologic convention.

The brain specimen was scanned on a whole-body human 7 Tesla (7 T) Siemens Magnetom MRI scanner (Siemens Healthineers, Erlangen, Germany) with the custom-built coil described above. We utilized a GRE sequence^19^ at 100 µm isotropic spatial resolution with the following acquisition parameters: TR = 40 msec, TE = 14.2 msec, bandwidth = 90 Hz/px, FA = 15°, 20°, 25°, 30°. Total scan time for each FA was 25:01:52 [hh:mm:ss], and each FA acquisition generated 1.98 TB of raw k-space data. To improve the signal-to-noise ratio (SNR) and optimise T_1_ modelling, we collected FLASH scans at four FAs (Fig. 3). Accounting for localizers, quality assurance (QA) scans, and adjustment scans, the total scan time was 100 hours and 8 minutes, and we collected nearly 7.92 TB of raw k-space data.

### MRI data reconstruction

The size of the k-space data exceeded the storage capacity of the RAID provided by the scanner’s image reconstruction computer. The image reconstruction also required more RAM than what was available. We therefore implemented software on the scanner to stream the data directly via TCP/IP to a server on an external computer added to the scanner network, which saved the data as they were received. Because of additional limitations related to the total size of the raw data for any single scan, as dictated by the imager RAID size, we also divided each acquisition into segments. The server on the external computer stored the data as they were acquired, creating date stamps for every k-space segment.

After the scan was completed, the streamed k-space data were transferred to a computational server where we ran custom software to stitch together the segments, reconstruct the images for each channel (via a 3D FFT on each volume per channel^20^), and combine the images derived from the 31 channels via the root-sum-of-squares of the signal magnitudes at each voxel. These signal magnitudes were channel-wise decorrelated using a covariance matrix of the channels’ thermal noise. The output from coil combination was the final acquired image (Videos 1, 2 and 3)^21^.

### MRI data processing

The acquired data underwent a series of processing steps, culminating in the creation of synthesized FLASH volumes, which are recomputed images incorporating all acquired measurements (Fig. 3; Videos 4, 5, 6, and 7)^21^. The volumes were estimated directly from the four FLASH acquisitions using the DESPOT1 algorithm^19,22^ with the program ‘mri_ms_fitparms’ distributed in FreeSurfer (http://surfer.nmr.mgh.harvard.edu)^23^ to quantify tissue properties independent of scanner and sequence types. This algorithm fits the tissue parameters (i.e. T_1_ and proton density) of the signal equation for the FLASH scan at each voxel using multiple input volumes. The volumes at the originally acquired TRs and FAs were then regenerated from the parameter maps by evaluating the FLASH signal equation. Of note, in this analysis we did not correct for B1 transmit inhomogeneities as we did not acquire the B1 transmit field map. Nevertheless, this method represents a disciplined approach to combining the volumes acquired with different FAs, while also providing a way to generate a variety of contrasts for distinguishing between multiple tissue classes.

**Fig. 4:**
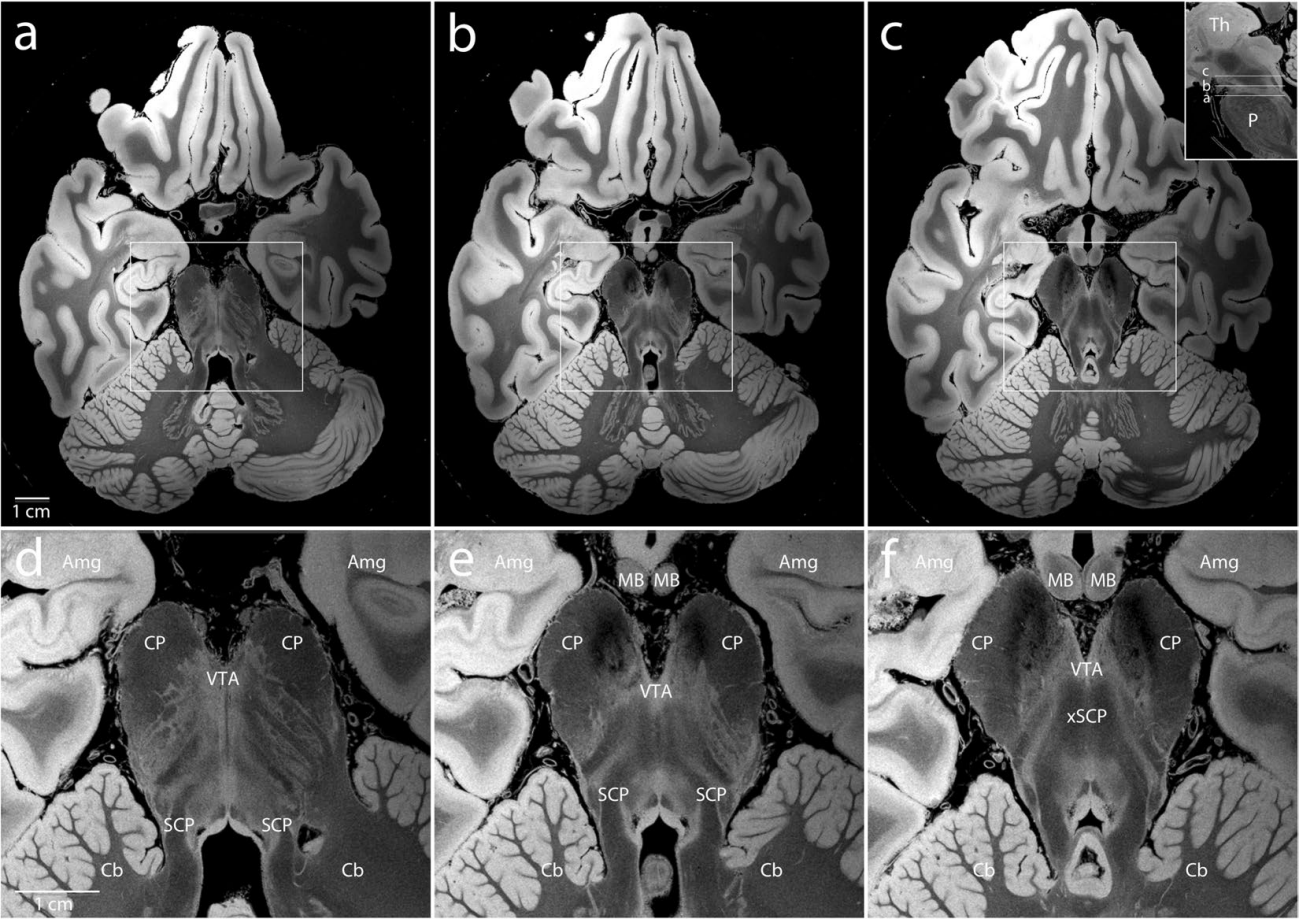
Delineation of brainstem neuroanatomy. Representative axial sections from the synthesized FLASH25 volume are shown at the level of the rostral pons and caudal midbrain (**a-c**, see inset in panel c). Zoomed views of the brainstem, medial temporal lobe, and anterior cerebellum (within the white rectangles in **a**–**c**) are shown in the bottom row (**d**–**f**). The anatomic detail that can be visualized in this *ex vivo* 100 μm resolution MRI dataset is beyond that which can be seen in typical *in vivo* MRI datasets. All images are shown in radiologic convention. Neuroanatomic abbreviations: Amg = amygdala; Cb = cerebellum; CP = cerebral peduncle; MB = mammillary body; P = pons; SCP = superior cerebellar peduncle; VTA = ventral tegmental area; xSCP = decussation of the superior cerebellar peduncle; Th = thalamus.

**Fig. 5:**
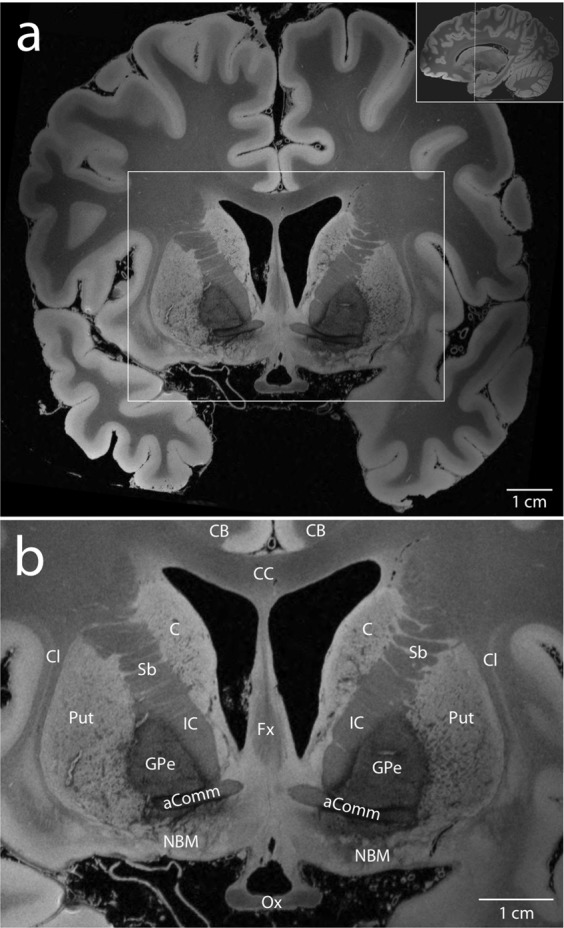
Delineation of basal ganglia and basal forebrain neuroanatomy. A representative coronal section from the synthesized FLASH25 volume is shown in the plane of the anterior commissure (aComm; see inset in **a**). A zoomed view of the basal ganglia and basal forebrain (within the white rectangle in **a**) is shown in (**b**). The anatomic detail that can be visualized in this *ex vivo* 100 μm resolution MRI dataset is beyond that which can be seen in standard *in vivo* MRI datasets. Neuroanatomic abbreviations: C = caudate; CB = cingulum bundle; CC = corpus callosum; Cl = claustrum; Fx = fornix; GPe = globus pallidus externa; IC = internal capsule; NBM = nucleus basalis of Meynert; Ox = optic chiasm; Put = putamen; Sb = striatal bridges.

In principle, a volume with any TR and FA combination could be synthesized. These synthesized volumes are created from all the acquired data and therefore have better SNR than the individually acquired input volumes. We choose to release the 25 degree synthetic volume (FLASH25) as it has, on average, maximal SNR and the best apparent contrast for cortical and subcortical structures^9^. Representative images from the FLASH25 volume are shown in Figs 4, 5, and Supplementary Fig. 2.

Of note, *ex vivo* MRI of the fixed human brain yields a different contrast than *in vivo* MRI, mainly from a shortened T_1_, but also from a decrease in T_2_^*^, both of which are related to formalin fixation^24^. The predominant source of signal contrast in *ex vivo* MRI is likely myelin^25^ and/or iron^26^. Specifically, myelin appears to be a source of T_1_ contrast, while cortical iron appears to be a source of T_2_^*^ contrast^27^.

### Coregistration of the dataset to standard stereotactic space

The dataset was spatially normalized into the MNI ICBM 2009b NLIN ASYM template^28^ (Supplementary Fig. 3a). This template constitutes the newest version of the “MNI space” and is considered a high-resolution version of MNI space because it is available at 0.5 mm isotropic resolution. To combine structural information present on T_1_ and T_2_ versions of the template, we created a joint template using PCA, as previously described^29^. The four synthesized FLASH volumes (FLASH15, FLASH20, FLASH25, and FLASH30) were downsampled to isotropic voxel-sizes of 0.5 mm for spatial normalization and initially registered into template space in a multispectral approach using Advanced Normalization Tools (ANTs; http://stnava.github.io/ANTs/)^30^. This multispectral approach simultaneously accounts for intensity data in all four volumes. The initial normalization was performed in four stages (rigid body, affine, whole brain SyN and subcortically focused SyN) as defined in the “effective: low variance + subcortical refine” preset implemented in Lead-DBS v2.0 software (www.lead-dbs.org)^31^. Lead-DBS is a MATLAB-based, version-controlled software package whose code is available at GitHub (www.github.com/netstim/leaddbs).

To refine the warp, we introduced fiducial regions of interest (ROI) iteratively using a tool developed for this task (available within Lead-DBS). Specifically, we manually drew line and point fiducial markers in both native and template spaces (Supplementary Fig. 3b). In addition, we manually segmented four structures in native space (subthalamic nucleus, internal and external pallidum and red nucleus). The three types of fiducials (line ROI, spherical ROI and manual segmentations of key structures) were then added as “spectra” in subsequent registration refinements (Supplementary Fig. 3c). Thus, the final registration consisted of a large number of pairings between native and template space (the first four being the actual anatomical volumes, the subsequent ones being manual segmentations and paired helper fiducials). To achieve maximal registration precision, the warp was refined in 132 iterations with extensive manual expert interaction, each refinement continuing directly from the last saved state. We used linear interpolation to create the normalized files in the data release^21^. Because the registration required manual segmentations and fiducial markers created by a neuroanatomy expert (A.H.), the registration is not replicable using code. Nevertheless, segmentations and fiducial markers, as well as final warp-files (which can be applied to the dataset using the ANTs package), are available upon request.

## Data Records

The native space FA25° acquisition and synthesized FLASH25 volume are available for download at the Dryad Digital Repository^21^ and OpenNeuro^32^. Additional synthesized volumes are available upon request to the corresponding author. Axial, coronal, and sagittal videos of the native space FA25° acquisition (Videos 1, 2, and 3) and synthesized FLASH25 volume (Videos 4, 5, 6, and 7) are also available at the Dryad Digital Repository^21^ and OpenNeuro^32^. The synthesized FLASH25 volume is available for interactive, online viewing at https://histopath.nmr.mgh.harvard.edu/100micronMRI. The normalized FLASH25 volume in standard stereotactic space is available at the Dryad Digital Repository^21^, OpenNeuro^32^, and is hosted on www.lead-dbs.org (preinstalled as part of the Lead-DBS software package).

To view the nifti volumes released with this dataset, we recommend using the FreeView viewer, which can be downloaded at: https://surfer.nmr.mgh.harvard.edu/fswiki/UpdateFreeview. FreeView is compatible with Linux, Mac, and Windows (via VirtualBox) operating systems. FreeView provides an opportunity to visualize the data in 3-dimensions, while also performing basic measurements and segmentations, and taking screenshots. With respect to system requirements, we recommend at least 8 GB of RAM and a processor speed of 2 GHz. For users whose computing hardware does not support visualization of the 100 micron resolution volumes, we downsampled the acquired FA25° volume, synthesized FLASH25 volume, and MNI-coregistered FLASH25 volume to 200 micron and 500 micron resolution. All downsampled volumes are available at Dyrad Digital Repository^21^ and OpenNeuro^32^. Users can also downsample the 100 micron volumes to their preferred spatial resolution using commands provided in the “README_code” file on OpenNeuro^32^.

To optimize the potential applications of this dataset, we also release the acquired volumes for all four flip angles (FA15°, FA20°, FA25°, FA30°) as source data on Open Neuro^32^, along with code to process the source data. With this code, users can create derivative volumes according to their preferences. We also provide the full set of sequence parameters from this scan on Dryad Digital Repository^21^ and OpenNeuro^32^.

## Technical Validation

### Coil signal-to-noise ratio (SNR) measurements

The receive coil has a Q_UL_/Q_L_ ratio that ranged from 6 in the top half elements to 8 in the bottom half elements due to larger coil diameter. The S_12_ coupling between neighbouring elements, measured with all other coils active detuned, ranged from −10.9 to −24 dB. All individual elemnts had S_11_ < −20 dB and active detuning of >30 dB. We evaluated the performance of the transmit coil by examining the B_1_^+^ profile^14^, which shows the efficiency throughout the entire spatial distribution of the brain specimen. The efficiency was greatest in the center of the specimen and fell off gradually towards the edges, as expected for a whole brain specimen at 7 T.

**Fig. 6:**
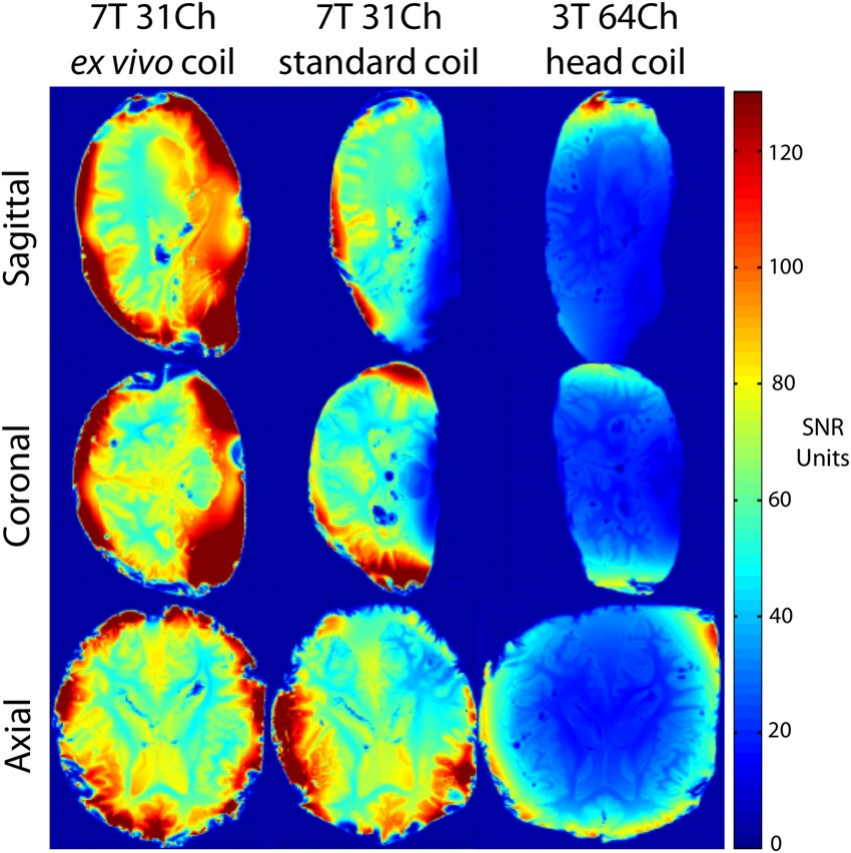
Signal-to-noise ratio (SNR) analysis of coil performance. Representative SNR maps are shown in the sagittal (top row), coronal (middle row) and axial (bottom row) planes for a test brain sample immersed in periodate-lysine-paraformaldehyde. The maps demonstrate an SNR gain of 1.6-fold for the 31-channel 7 Tesla (7 T) *ex vivo* coil (left column) compared to the 31-channel 7 T standard coil (middle column), and a gain of 3.3-fold compared to the 64-channel 3 T head coil (right column). The noise coupling between channels was 11% for the 31-channel *ex vivo* coil array, a 2-fold improvement relative to our previous array^18^.

We compared the SNR of the 31-channel *ex vivo* array to that of a standard 31-channel 7 T head coil and a 64-channel 3 T head coil. SNR maps were computed following the method of Kellman & McVeigh^33^. We calibrated the voltage required for a 180° pulse using a B_1_^+^ map (estimated with the AFI method)^34^ with an ROI of 3-cm diameter at the center of the brain. We estimated array noise covariance from thermal noise data acquired without RF excitation. The SNR gain with the 31-channel *ex vivo* array was 1.6-fold versus the 31-channel 7 T standard coil and 3.3-fold versus the 64-channel 3 T head array (Fig. 6). The noise coupling between channels was 11% for the 31-channel *ex vivo* array, a 2-fold improvement relative to our previous array^18^.

### Coregistration accuracy

**Fig. 7:**
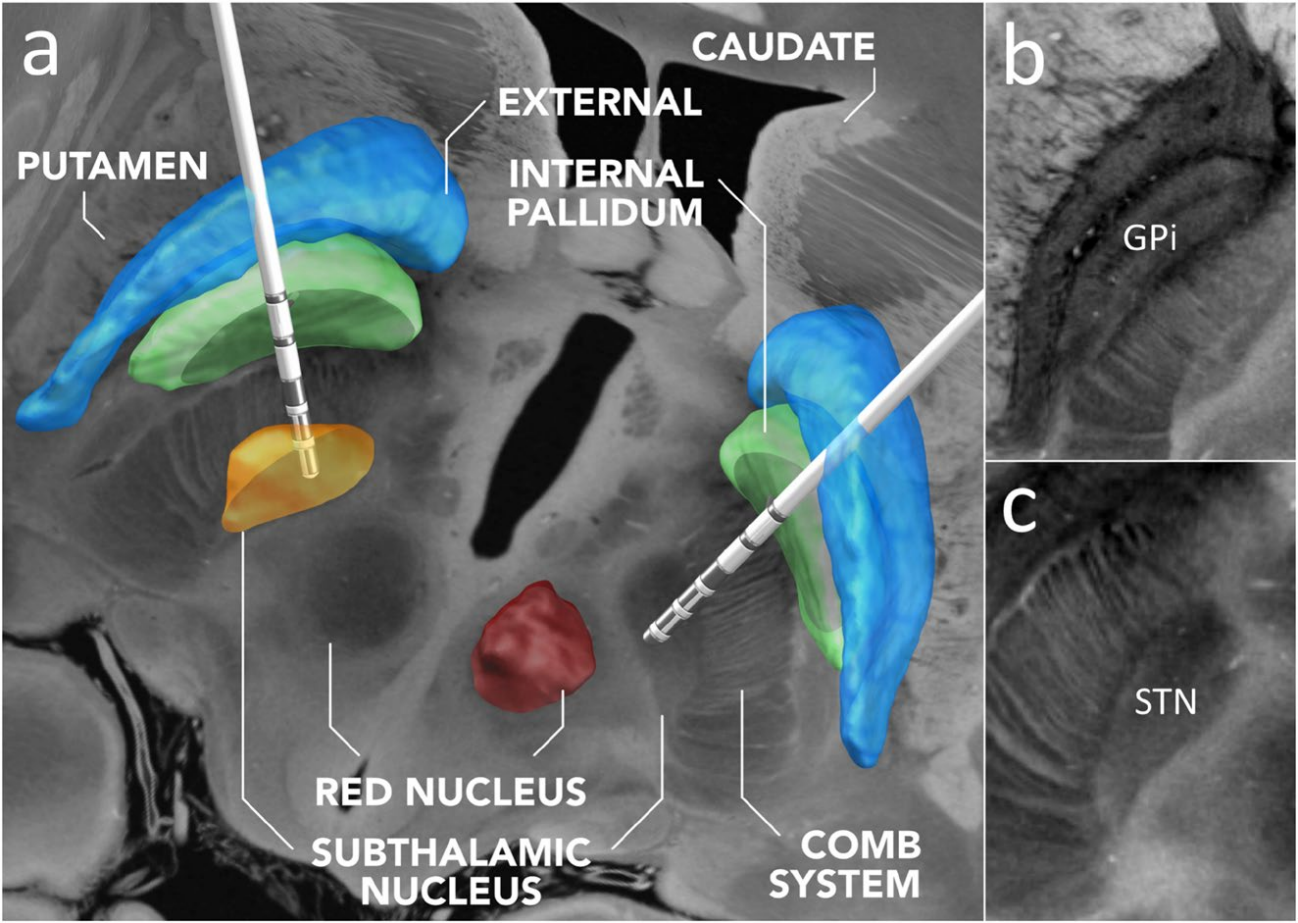
Normalization of the *ex vivo* MRI dataset into standard stereotactic space and integration into the Lead-DBS software platform. (**a)** Exemplary use-case of the normalized FLASH25 volume in deep brain stimulation (DBS). DBS electrodes are visualized for a hypothetical patient using Lead-DBS software (https://www.lead-dbs.org)^31^. An axial image from the normalized scan, at the level of the rostral midbrain, is shown as a backdrop, with 3D-structures defined by the DISTAL atlas^35^ (right subthalamic and left red nucleus hidden for optimal visualization of the underlying anatomy). Panels (**b**) and (**c**) show zoomed views of key DBS target regions: the left globus pallidus interna (GPi in **b**) and subthalamic nucleus (STN in **c**).

We assessed the neuroanatomic accuracy of the final registration results (i.e. the fit between structures on the normalized FLASH volumes versus the high-resolution MNI template) by visual inspection using a tool specifically designed for this task (implemented in Lead-DBS). An example of this visual inspection assessment for the subthalamic nucleus and globus pallidus interna is provided in Supplementary Fig. 4. The normalized FLASH25 volume is distributed pre-installed within Lead-DBS software and can be selected for visualization in the 3D viewer (www.lead-dbs.org). Figure 7 shows an example of deep brain stimulation electrode reconstructions in a hypothetical patient being treated for Parkinson’s Disease.

## Supplementary information

Supplementary Material.

## Data Availability

Neuroimaging data were processed using standard processing pipelines in FreeSurfer v6.0^23^ (http://surfer.nmr.mgh.harvard.edu, https://github.com/freesurfer/freesurfer). All code used for registration of volumes into standard stereotactic space is available within the open-source Lead-DBS v2.0 software^31^ (https://github.com/leaddbs/leaddbs). Because registration involved multiple manual user interface steps, no ready-made code is provided, but the process can be readily reproduced with the provided data and software.
