# Supplementary material for: 7 Tesla MRI of the ex vivo human brain at 100 micron resolution
Source: Sci Data. 2019 Oct 30;6:244. doi: 10.1038/s41597-019-0254-8 (PMC6821740; doi:10.1038/s41597-019-0254-8)
Supplement: Supplementary file 1 — Supplementary Material. [file 41597_2019_254_MOESM1_ESM.pdf]

SUPPLEMENTARY MATERIAL

7 Tesla MRI of the *ex vivo* human brain at 100 micron resolution

Brian L. Edlow[1,2], Azma Mareyam[2], Andreas Horn[3], Jonathan R. Polimeni[2], Thomas Witzel[2], M. Dylan Tisdall[4], Jean Augustinack[2], Jason P. Stockmann[2], Bram R. Diamond[2], Allison Stevens[2], Lee S. Tirrell[2], Rebecca D. Folkerth[5], Lawrence L. Wald[2], Bruce Fischl[2,*] & Andre van der Kouwe[2,*]

* co-senior authors

1. Center for Neurotechnology and Neurorecovery, Massachusetts General Hospital, Department of Neurology, Boston, MA 02114, USA

2. Athinoula A. Martinos Center for Biomedical Imaging, Massachusetts General Hospital, Department of Radiology, Charlestown, MA 02129, USA

3. Movement Disorders & Neuromodulation Section, Department for Neurology, Charité – University Medicine Berlin, Germany

4. Radiology, Perelman School of Medicine, University of Pennsylvania, Philadelphia, PA 19104, USA

5. City of New York Office of the Chief Medical Examiner, and New York University School of Medicine, New York, NY 10016, USA

corresponding author: Brian L. Edlow, M.D. (bedlow@mgh.harvard.edu)

Methods Overview

1) Brain specimen fixation and preparation (adapted from Edlow et al., *Journal of Neurotrauma*, 2018)[1]:
    a. Place brain specimen in a 10 x 10 x 10 inch bucket that accommodates a large volume of neutral buffered 10% formalin.
    b. Fill the bucket with enough neutral buffered 10% formalin to completely surround the specimen.
    c. Suspend the brain to ensure that fixative surrounds the brain and that no brain surfaces are compressed against the base or sides of the bucket.
        i. If the basilar artery is intact, hang the specimen via a string placed underneath the basilar artery.
        ii. If the basilar artery is not intact, hang the specimen using a bouffant cap (i.e. hairnet). Use string to hang the hairnet/bouffant and use an elastic band to hold the hairnet.
    d. Neutral buffered 10% formalin must be changed *at a minimum* every week for one month to ensure adequate fixation.
2) Brain specimen scanning
    a. See “100_Micron_MRI_Scan_Protocol.pdf” on Dryad Digital Repository[2] and OpenNeuro[3].
3) MRI data processing
    a. see “README_code.pdf” on OpenNeuro[3].
4) MRI data viewing

a. To view the nifti volumes released with this dataset, we recommend using FreeView, which can be downloaded at: https://surfer.nmr.mgh.harvard.edu/fswiki/UpdateFreeview. FreeView is compatible with Linux, Mac, and Windows (via VirtualBox) operating systems.

5) MRI data coregistration to MNI space

   a. Coregistration can be performed using Lead-DBS v2.0 software (www.lead-dbs.org[4]). Lead-DBS is a MATLAB-based software package whose code is available at GitHub (www.github.com/netstim/leaddbs).

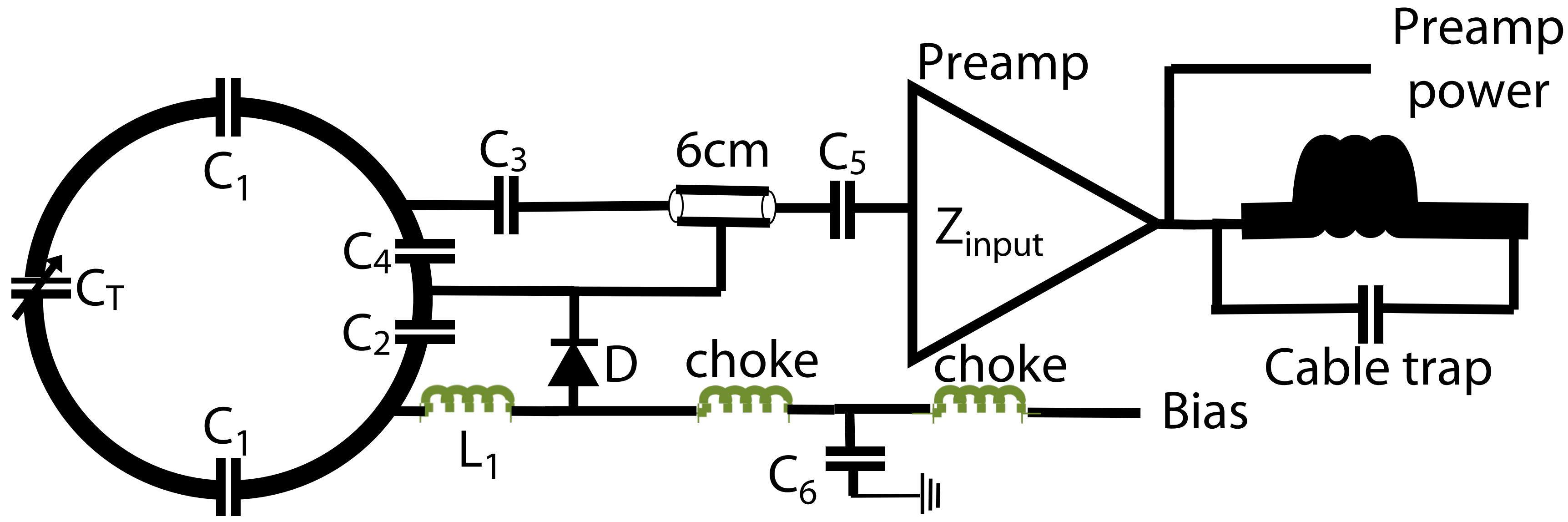

Supplementary Figure 1. Circuit diagram of each coil element. All elements were constructed using 16 AWG wire loops[5], each with four or five evenly spaced capacitors. All elements were tuned to 297.2 MHz and matched to a loaded impedance of 75 Ω to minimize preamplifier noise. Preamplifier decoupling was achieved with a cable length of 6 cm. Preamplifiers were placed directly on the coil elements, yielding a substantial reduction in cable losses compared to a previous 30-channel *ex vivo* brain array[6].

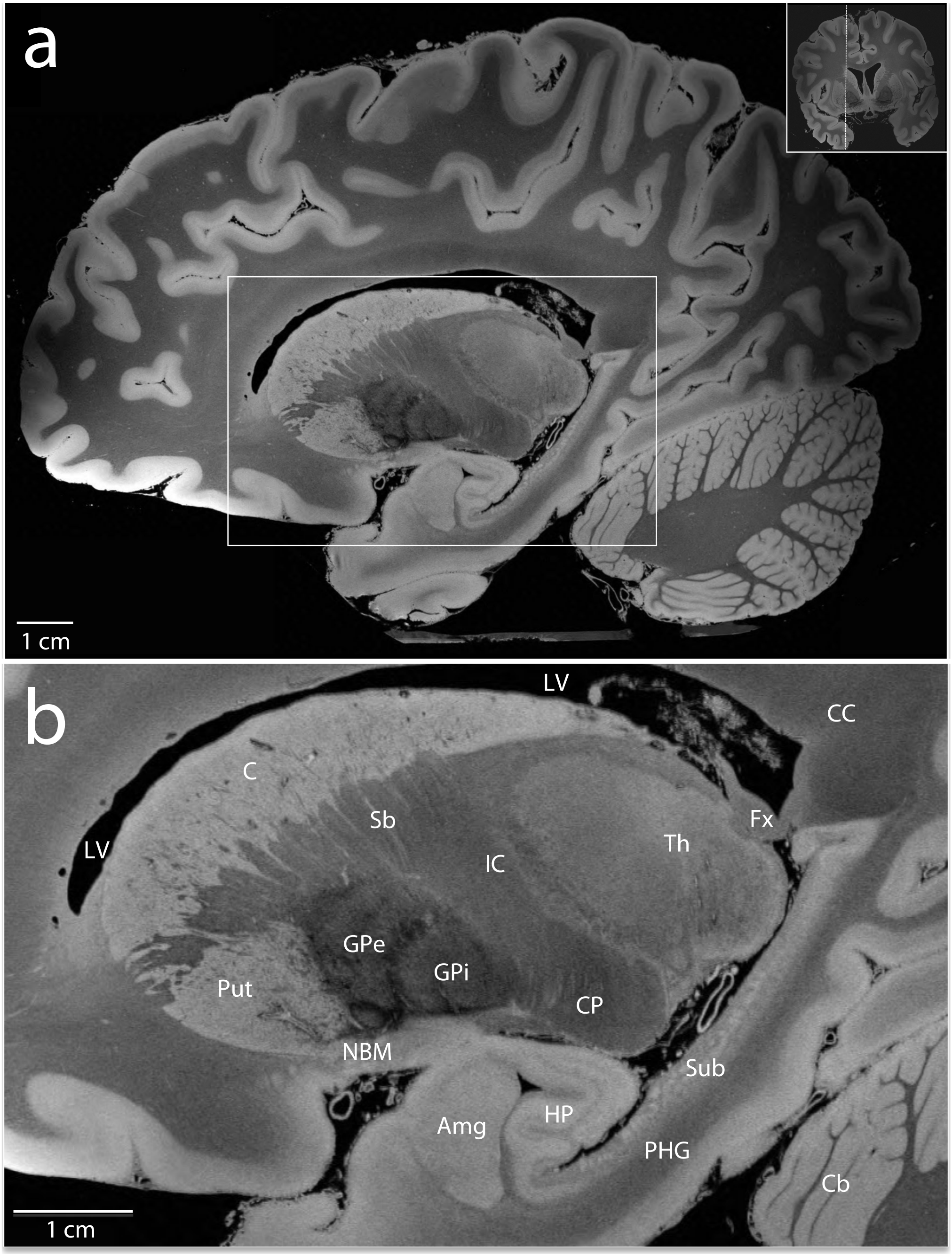

Supplementary Figure 2. Delineation of basal ganglia, diencephalon and medial temporal neuroanatomy. A representative sagittal section from the synthesized FLASH25 volume is shown in the plane of the striatum (see inset in **a**). A zoomed view of the striatum, amygdala (Amg) and hippocampus (HP) (within the white rectangle in **a**) is shown in **b**. The anatomic detail that can be visualized in this *ex vivo* 100 μm resolution MRI dataset is beyond that which can be seen in typical *in vivo* MRI datasets. Neuroanatomic abbreviations: C = caudate; Cb = cerebellum; CC = corpus callosum; CP = cerebral peduncle; Fx = fornix; GPe = globus pallidus externa; GPi = globus pallidus interna; IC = internal capsule; LV = lateral ventricle; NBM = nucleus basalis of Meynert; PHG = parahippocampal gyrus; Put = putamen; Sb = striatal bridges; Sub = subicular cortices; Th = thalamus.

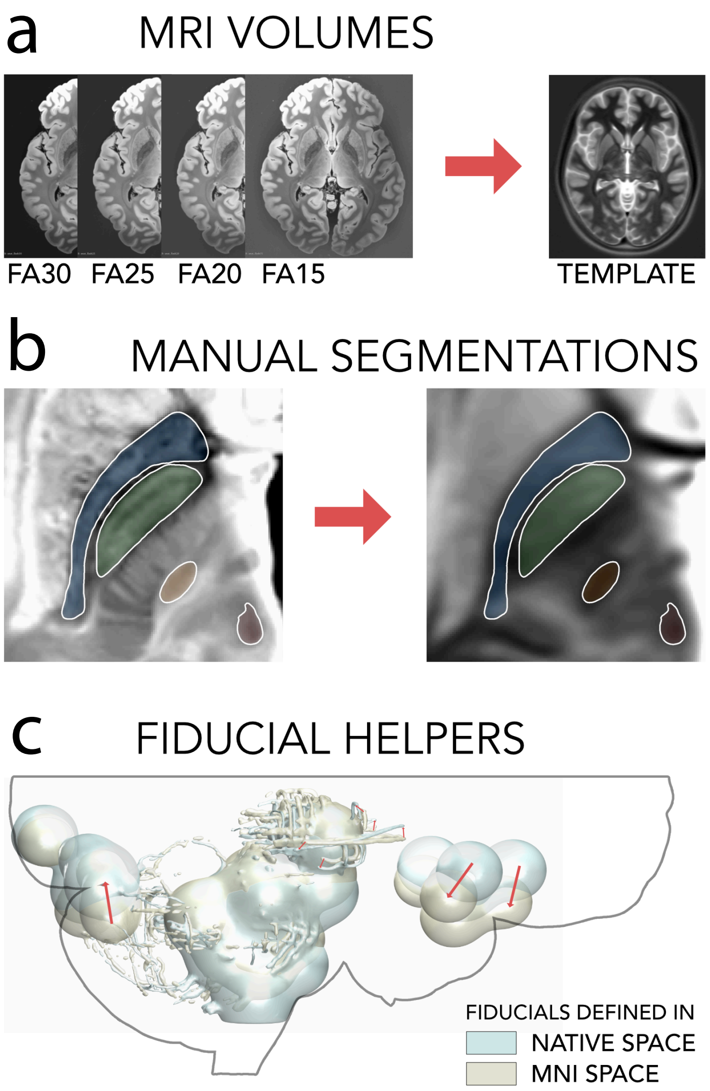

Supplementary Figure 3. Coregistration to standard stereotactic space. (**a**) We performed a multispectral warp between FLASH15-30 volumes and the PCA template of the ICBM 2009b NLIN ASYM space[7,8], which built the basis of the iterative registration process. (**b**) In addition, we manually segmented key subcortical structures – the subthalamic nucleus (orange), red nucleus (red), internal (green) and external (blue) pallidum – in native space and paired them with corresponding structures in MNI space, as defined by the DISTAL atlas[9]. (**c**) Additionally, we manually introduced line and smoothed sphere fiducial markers into native and MNI spaces, and we paired them in the registration process. Registration was performed in 132 iterations, and fiducial corrections (shown in (**c**)) were introduced iteratively to further optimize fit.

SUBTHALAMIC NUCLEUS

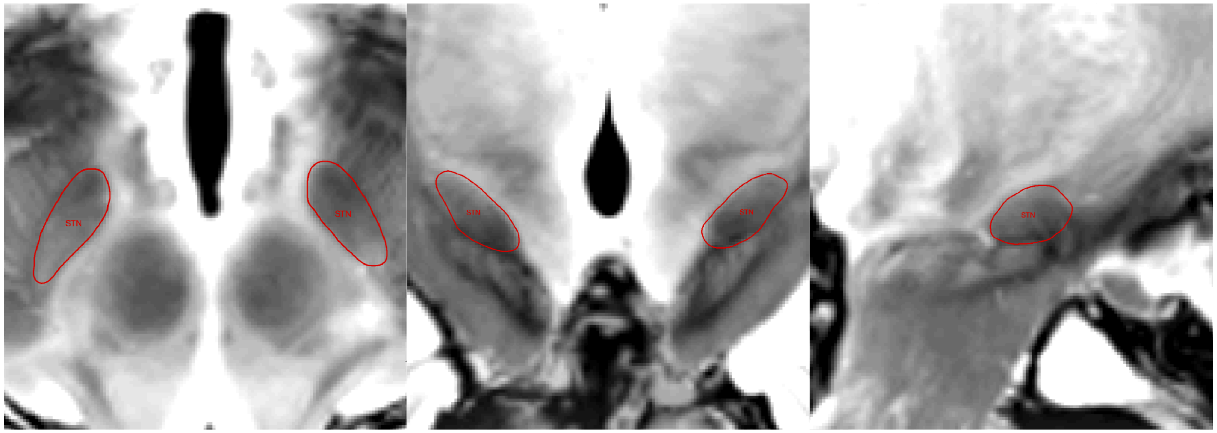

INTERNAL PALLIDUM

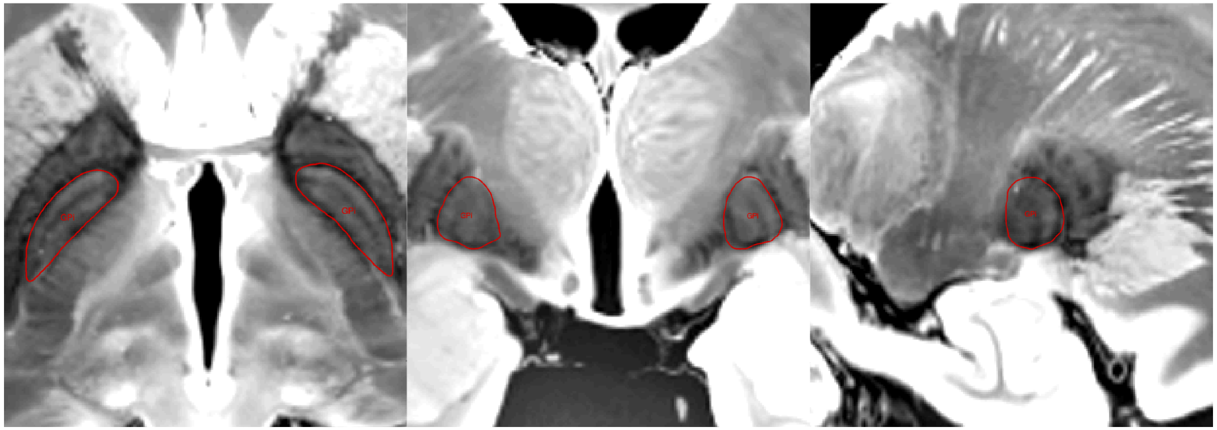

Supplementary Figure 4. Neuroanatomic accuracy of the coregistration to stereotactic space. We show the registration fit for subcortical structures that are clinically relevant to deep brain stimulation: subthalamic nucleus (STN) and internal pallidum (GPi). Red lines show the boundaries of these structures defined by the DISTAL atlas[9]. The normalized FLASH25 volume is shown in the background (left column = axial, middle column = coronal, right column = sagittal views).

Supplementary References

1 Edlow, B. L. *et al.* Multimodal Characterization of the Late Effects of Traumatic Brain Injury: A Methodological Overview of the Late Effects of Traumatic Brain Injury Project. *J Neurotrauma* **35**, 1604-1619, doi:10.1089/neu.2017.5457 (2018).

2 Edlow, B. L. *et al.* Data from: 7 Tesla MRI of the *ex vivo* human brain at 100 micron resolution. *Dryad Digital Repository*, https://doi.org/10.5061/dryad.119f80q (2019).

3 Edlow, B. L. *et al.* Data from: 7 Tesla MRI of the *ex vivo* human brain at 100 micron resolution. *OpenNeuro*, https://doi.org/10.18112/openneuro.ds002179.v1.1.0 (2019).

4 Horn, A. *et al.* Lead-DBS v2: Towards a comprehensive pipeline for deep brain stimulation imaging. *Neuroimage* **184**, 293-316, doi:10.1016/j.neuroimage.2018.08.068 (2019).

5 Keil, B. *et al.* Size-optimized 32-channel brain arrays for 3 T pediatric imaging. *Magn Reson Med* **66**, 1777-1787, doi:10.1002/mrm.22961 (2011).

6 Mareyam, A., Polimeni, J. R., Alagappan, V., Fischl, B. & Wald, L. L. A 30 channel receive-only 7T array for ex vivo brain hemisphere imaging. *International Society for Magnetic Resonance in Medicine.* Abstract #106 (2009).

7 Horn, A. PCA MNI 2009b NLIN template. https://doi.org/10.6084/m9.figshare.4644472.v2 (2017).

8 Fonov, V. S., Evans, A. C., McKinstry, R. C., Almli, C. R. & Collins, D. Unbiased nonlinear average age-appropriate brain templates from birth to adulthood. *Neuroimage* **47**, S102 (2009).

9 Ewert, S. *et al.* Toward defining deep brain stimulation targets in MNI space: A subcortical atlas based on multimodal MRI, histology and structural connectivity. *Neuroimage* **170**, 271-282, doi:10.1016/j.neuroimage.2017.05.015 (2018).
